# Identification of DNA damage repair-related genes in sepsis using bioinformatics and machine learning: An observational study

**DOI:** 10.1097/MD.0000000000041354

**Published:** 2025-01-31

**Authors:** Jin Gu, Dong-Fang Wang, Jian-Ying Lou

**Affiliations:** a Department of Hepatobiliary and Pancreatic Surgery, The Second Affiliated Hospital, School of Medicine, Zhejiang University, Hangzhou, China; b Trauma Center/Department of Emergency and Traumatic Surgery, Tongji Hospital, Tongji Medical College, Huazhong University of Science and Technology, Wuhan, China.

**Keywords:** bioinformatics, diagnostic biomarkers, DNA damage repair, immune infiltration, sepsis

## Abstract

Sepsis is a life-threatening disease with a high mortality rate, for which the pathogenetic mechanism still unclear. DNA damage repair (DDR) is essential for maintaining genome integrity. This study aimed to explore the role of DDR-related genes in the development of sepsis and further investigated their molecular subtypes to enrich potential diagnostic biomarkers. Two Gene Expression Omnibus datasets (GSE65682 and GSE95233) were implemented to investigate the underlying role of DDR-related genes in sepsis. Three machine learning algorithms were utilized to identify the optimal feature genes. The diagnostic value of the selected genes was evaluated using the receiver operating characteristic curves. A nomogram was built to assess the diagnostic ability of the selected genes via “rms” package. Consensus clustering was subsequently performed to identify the molecular subtypes for sepsis. Furthermore, CIBERSORT was used to evaluate the immune cell infiltration of samples. Three different expressed DDR-related genes (GADD45A, HMGB2, and RPS27L) were identified as sepsis biomarkers. Receiver operating characteristic curves revealed that all 3 genes showed good diagnostic value. The nomogram including these 3 genes also exhibited good diagnostic efficiency. A notable difference in the immune microenvironment landscape was discovered between sepsis patients and healthy controls. Furthermore, all 3 genes were significantly associated with various immune cells. Our findings identify potential new diagnostic markers for sepsis that shed light on novel pathogenetic mechanism of sepsis and, therefore, may offer opportunities for potential intervention and treatment strategies.

## 1. Introduction

Sepsis is a fatal syndrome caused by a dysregulated host immunological response to infection, which is usually linked to life-threatening multisystemic dysfunction.^[1,2]^ Some novel treatment strategies are used in clinical settings to stop disease progression, including early fluid resuscitation, use of antibiotics, blood purification, and extracorporeal membrane oxygenation.^[3–5]^ However, long-term ICU stays are increasing, and the mortality rate of sepsis remains high. Sophisticated factors contribute to the pathogenesis of sepsis, including unbalanced immune homeostasis, cell death, increased inflammation response, and excessive molecular regulation.^[6]^ Timely diagnosis and treatment for those patients associated with an increased mortality risk are essential for a better prognosis.^[7]^ Sepsis biomarkers,^[8,9]^ such as C-reactive protein, procalcitonin, and lactate, are used to predict the progression of the disease in patients. However, due to their limited precision and sensitivity, these markers are unreliable predictive tools sepsis individuals. Therefore, there is a need to explore the mechanism of sepsis comprehensively and search for novel diagnostic and therapeutic targets.

It has been widely accepted that sepsis is characterized by a significant inflammatory response to infection.^[10]^ Reactive oxygen species (ROS) and reactive nitrogen species (RNS) are produced by activated neutrophils and macrophages during the inflammatory response.^[11]^ ROS and RNS products can lead to reversible or irreversible chemical changes in proteins, lipids, and DNA, finally resulting in diminished biochemical functions.^[12]^ ROS and RNS can induce adducts to DNA, contributing to DNA fragmentation.^[13]^ The most significant damage to DNA includes single-strand breaks and double-strand breaks. The DNA repair mechanism is activated upon recognition of DNA damage, which attempts to repair the DNA of the cell. DNA damage repair (DDR) system has been reported to include the following 8 pathways: mismatch repair, base excision repair, nucleotide excision repair, homologous recombination repair, checkpoint factors, nonhomologous end-joining, Fanconi anemia, and translesion DNA synthesis.^[14]^ Studies have revealed that the DDR system plays an important role in tumorigenesis,^[15]^ neurodegenerative disorders,^[16]^ immune deficiencies and infertility, stem cell dysfunction, cardiovascular disease, and metabolic syndrome.^[17]^ However, studies elucidating the DDR gene’s involvement in sepsis remain scarce. Therefore, it is imperative to uncover the roles of DDR in sepsis.

Using bioinformatics tools, many effective diagnostic and therapeutic targets for sepsis have been identified. Machine learning together with bioinformatics can transform biomedical data into useful knowledge. It has been successfully used in terms of accuracy and speed to address issues involving biology and medicine.^[18]^ In this study, we extracted the sepsis datasets from the Gene Expression Omnibus database and investigated the role of DDR-related genes with the evolution of sepsis and detected their molecular subtypes to extend the variety of diagnostic biomarkers. This study provides new ideas and targets for the intervention and treatment of sepsis.

## 2. Methods

### 2.1. Data acquisition and processing

The GSE65682 and GSE95233 datasets were obtained from the Gene Expression Omnibus database (https://www.ncbi.nlm.nih.gov/geo/). The GSE65682 dataset included 40 healthy controls and 760 septic patients. The GSE95233 dataset included 22 healthy controls and 51 septic patients. Only the gene expression data of the blood sample collected at admission to the intensive care unit was used for analysis in this study. The gene probe was transformed into gene symbol by using the corresponding annotation profile in each dataset. We used “limma” package in R software (version 4.2.1) to quartile normalized for all gene expression values and generate normally distributed expression. For multiple same probes, the finial gene expression value was determined by calculating the average expression value. DDR-gene list containing 557 genes was retrieved from relevant gene lists, including Molecular Signatures Database from the Broad Institute (https://www.gsea-msigdb.org/gsea/msigdb/) and literature.^[19–21]^ Finally, 384 DDR genes detected in all datasets were analyzed in this study. Our study was exempted from ethical review. This article does not contain any studies with human or animal subjects performed by any of the authors.

### 2.2. Differentially EXPRESSED GENES SCREENING

Differentially expressed genes (DEGs) analysis between sepsis and healthy samples in GSE65682 was screened using the “limma” package, with |logFC| > 0.5 and adjust *P* < .05 set as cutoff criteria to screen DEGs. DDR-related DEGs were identified by intersecting the DEGs with the DDR-related genes and then visualized using R packages (pheatmap, dplyr, ggplot2, and ggrepel) to plot heatmaps and volcano plots, respectively.

### 2.3. Function enrichment analysis

Gene Ontology (GO) analysis and Kyoto Encyclopedia of Genes and Genomes (KEGG) analysis were completed to determine the potential functions and pathways of DDR-related DEGs using the “clusterProfiler” package, with *P* < .05 considered to be the cutoff value.

We then analyzed the upregulated and downregulated pathways in septic patients relative to healthy controls. We used the HALLMAKER gene set from the Molecular Signatures Database, which included 50 enriched pathways. GSVA was performed for the enrichment results using “GSVA” package.

### 2.4. Optimal feature genes selection

The optimal feature genes were determined using least absolute shrinkage and selection operator (LASSO) regression, support vector machine (SVM), and random forest graph analysis. The LASSO regression algorithm was carried out using the “glmnet” package in R, and the optimal penalty parameter was set based a 10-fold cross-validation minimum.^[22]^ The SVM classifier found in the “e1071” package in R was used to determine the optimal feature genes. Furthermore, random forest graph analysis was performed using the “randomforest” package in R. Finally, we used the venn diagram to visualize the optimal feature genes common to the 3 machine learning methods.

### 2.5. Differential expression analysis and receiver operating characteristic curve validation

The GSE65682 and GSE95233 were used to validate the differential expression of the genes obtained by 3 machine learning algorithms analysis. The diagnostic value of the optimal feature genes for sepsis was analyzed using the “pROC” package in R. ROC curve and the area under the curve were calculated.

### 2.6. Nomogram construction

The nomogram was built by the “rms” package in R. “Points” indicate the scores of a candidate gene, and “Total Points” represents the sum of the above scores for all genes. The calibration curve and C-index were used to evaluate the performance of the nomogram and to visually reflect how well the predicted probabilities matched the observed probabilities, therefore demonstrating the important value of the DDR in sepsis.

### 2.7. CeRNA

A competing endogenous RNA (ceRNA) regulatory network refers to the entire regulatory network involving ceRNAs, which usually includes mRNA, miRNAs, and lncRNAs. We constructed the mRNA–miRNA–lncRNA regulatory network using the miRanda, miRDB, and TargetScan databases, which was subsequently visualized using Cytoscape.

### 2.8. Immune cell infiltration and clustering analysis

We performed immune infiltration analysis using CIBERSORT (https://cibersortx.stanford.edu/) to assess the profiles of 22 kinds of infiltrating immune cells according to the expression of samples in the GSE65682 dataset. Violin plots were performed using the “vioplot” package in R to visualize the variations in immune cell infiltration. Correlation analyses were performed to investigate the correlation between the expression of optimal feature genes and immune cells.

The identified diagnostic biomarkers were utilized for clustering analysis using the “ConsensusClusterPlus” package in R, with a maximum K of 9. Sepsis patients were classified into 2 molecular subtypes according to the optimal classification of K = 2. The “ggplot2” package in R was used to show the distribution pattern of sepsis and healthy samples in a PCA plot based on the immune cells proportion.

## 3. Results

### 3.1. Identification of DEGs in sepsis

**Figure 1.**
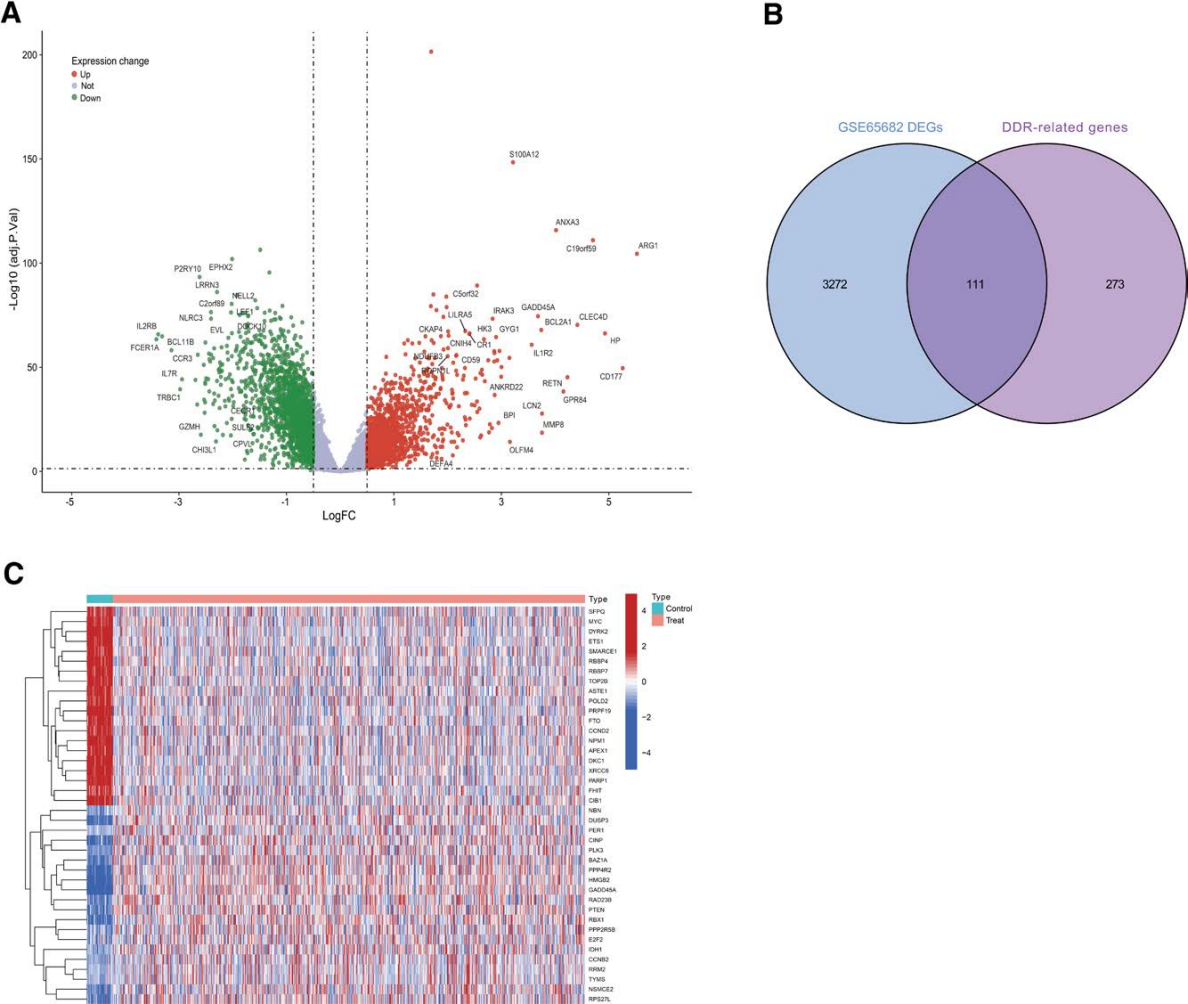
(A) Volcano plot of DEGs in the GSE65682 dataset between septic patients and healthy controls. (B) Venn plot of the DEGs and DNA damage repair (DDR)-genes. (C) Heatmap of top 20 differential genes (upregulated and downregulated). DDR = DNA damage repair, DEGs = differentially expressed genes.

A total of 3383 DEGs were identified in this study using the GSE65682 dataset (containing sample data from 40 healthy controls and 760 septic patients) based on the criteria of |logFC| > 0.5 and adjust *P* < .05, including 1528 upregulated genes and 1855 downregulated genes (Fig. 1A). After intersecting with the DDR-related genes, 111 DDR-related DEGs were obtained (Fig. 1B). The top 20 genes from the upregulated and downregulated genes were selected separately for the heatmap, with red indicating upregulated genes and blue indicating downregulated gene (Fig. 1C).

### 3.2. Functional enrichment analysis of DEGs

**Figure 2.**
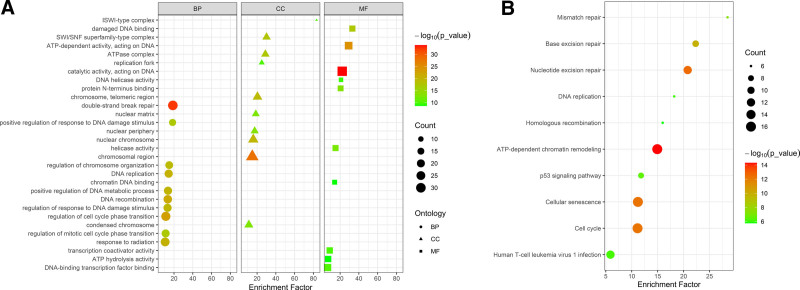
Functional enrichment analysis of DDR-related DEGs in septic samples. (A) The most significantly enriched GO terms of DDR-related DEGs. (B) KEGG pathway enrichment analysis of DDR-related DEGs. DDR = DNA damage repair, DEGs = differentially expressed genes, GO = Gene Ontology, KEGG = Kyoto Encyclopedia of Genes and Genomes.

To understand the biological functions and potential pathways of the 111 DDR-related DEGs, we performed functional enrichment analysis. The results of GO analysis indicated that the top 5 enriched biological processes were double-strand break repair, positive regulation of response to DNA damage stimulus, regulation of chromosome organization, DNA replication, and positive regulation of DNA metabolic process. The top 5 cellular components were ISWI-type complex, SWI/SNF superfamily-type complex, ATPase complex, replication fork, and chromosome, telomeric region. The top 5 enriched molecular functions were damaged DNA binding, ATP-dependent activity, acting on DNA, catalytic activity, acting on DNA, DNA helicase activity, and protein N-terminus binding (Fig. 2A).

KEGG analysis showed that the main enriched pathways were mismatch repair, base excision repair, nucleotide excision repair, DNA replication, and homologous recombination (Fig. 2B).

### 3.3. Identification of optimal feature genes and their mechanisms in sepsis

**Figure 3.**
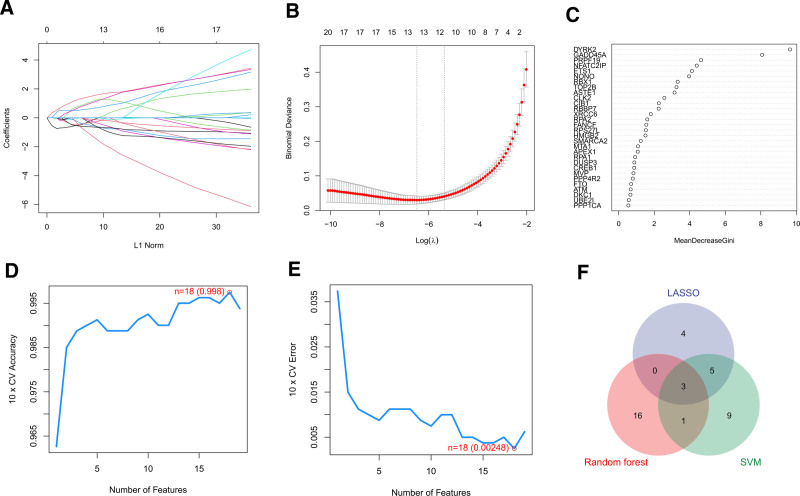
Feature genes selection via 3 machine language algorithms. (A and B) LASSO analysis to screen key DDR-related DEGs. (C) Random forest analysis of key DDR-related DEGs. (D and E) SVM algorithm to identify the optimal DDR-related DEGs. (F) Venn diagram showing 3 feature genes intersected by 3 algorithms. DDR = DNA damage repair, DEGs = differentially expressed genes, LASSO = least absolute shrinkage and selection operator, SVM = support vector machine.

To identify DDR-related biomarkers of sepsis, 3 machine learning algorithms were applied. Using the LASSO algorithm, 12 genes were identified as diagnostic biomarkers for sepsis (Fig. 3A and B). Using the random forest algorithm, 20 feature genes were identified for further analysis (Fig. 3C). Besides, the SVM algorithm was utilized to specialize the features of DDR-related DEGs to 18 variables (Fig. 3D and E). Three overlapping genes (GADD45A, HMGB2, and RPS27L) were finally identified among the 3 algorithms (Fig. 3F).

**Figure 4.**
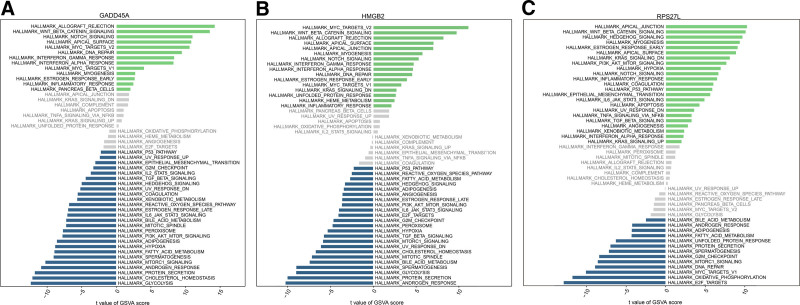
Single gene GSVA pathway analysis for (A) GADD45A; (B) HMGB2; (C) RPS27L.

To further explore the underlying molecular mechanisms of the selected DDR-related genes in sepsis using GSE65682 dataset, we then conducted an analysis using the GSVA approach. The results based on the HALLMAKER gene set are shown in Figure 4, where the obviously highly enriched pathway of the 3 gens was “WNT_BETA_CATENIN_SIGNALING.”

### 3.4. Validation of the selected DDR-related genes and effectiveness evaluation

**Figure 5.**
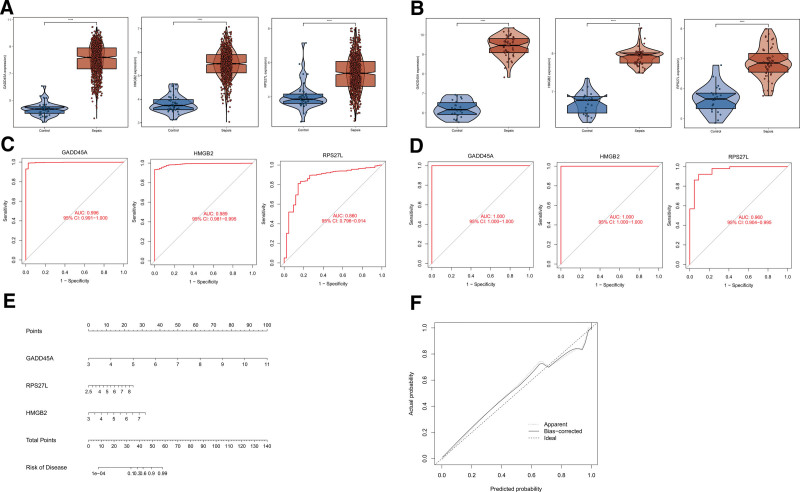
(A) Expression levels of GADD45A, HMGB2, and RPS27L in septic patients compared with healthy controls in the GSE65682 dataset. (B) Expression levels of GADD45A, HMGB2, and RPS27L in septic patients compared with healthy controls in the GSE95233 dataset. (C) ROC analysis of GADD45A, HMGB2, and RPS27L in the GSE65682 dataset. (D) ROC analysis of GADD45A, HMGB2, and RPS27L in the GSE95233 dataset. (E) Nomogram construction of 3 genes signature in the GSE65682 dataset. (F) Calibration curve plot for the nomogram. ROC = receiver operating characteristic.

We further analyzed the expression of GADD45A, HMGB2, and RPS27L in GSE65682 and GSE95233 datasets. Compared with healthy controls, the expression of GADD45A, HMGB2, and RPS27L were significantly up-regulated in septic patients compared with healthy controls (all *P* < .001) (Fig. 5A and B). Furthermore, ROC curve analysis was performed using samples from the healthy controls and septic patients in GSE65682 dataset as study samples and in GSE95233 as validation samples. The results suggested that these genes show superior representation for sepsis with the area under the curve values >0.85 (Fig. 5C and D).

To further assess the clinical applicability of the 3 genes signature, the nomogram was built based on the GSE65682 datasets (Fig. 5E). The calibration plots showed that the nomogram model composed of 3 genes, GADD45A, HMGB2, and RPS27L, exhibited good diagnostic prediction for sepsis (Fig. 5F).

### 3.5. ceRNA networks based on the selected DDR-related genes

**Figure 6.**
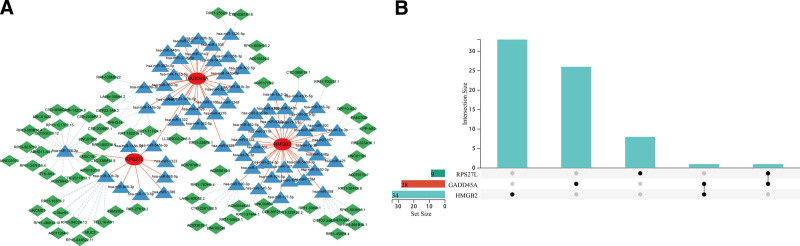
Construction of ceRNA network based on 3 marker genes. (A) ceRNA network based on 3 marker genes. (B) UpSet plot was utilized to present the interaction network of marker genes and miRNAs. ceRNA = competing endogenous RNA.

To explore the possible mechanisms involved in the dysregulation of selected genes, we constructed a ceRNA network based on 3 marker genes using the using the miRanda, miRDB, and TargetScan databases. The network included 144 nodes (3 marker genes, 69 miRNAs, and 72 lncRNAs), and the specific network is shown in Figure 6A. Furthermore, 2 miRNAs were able to control at least 2 mRNA, including hsa-miR-4328 (controlling GADD45A and HMGB2) and hsa-miR-548a-3p (controlling GADD45A and RPS27L) (Fig. 6B).

### 3.6. Analysis of immune microenvironment landscape

**Figure 7.**
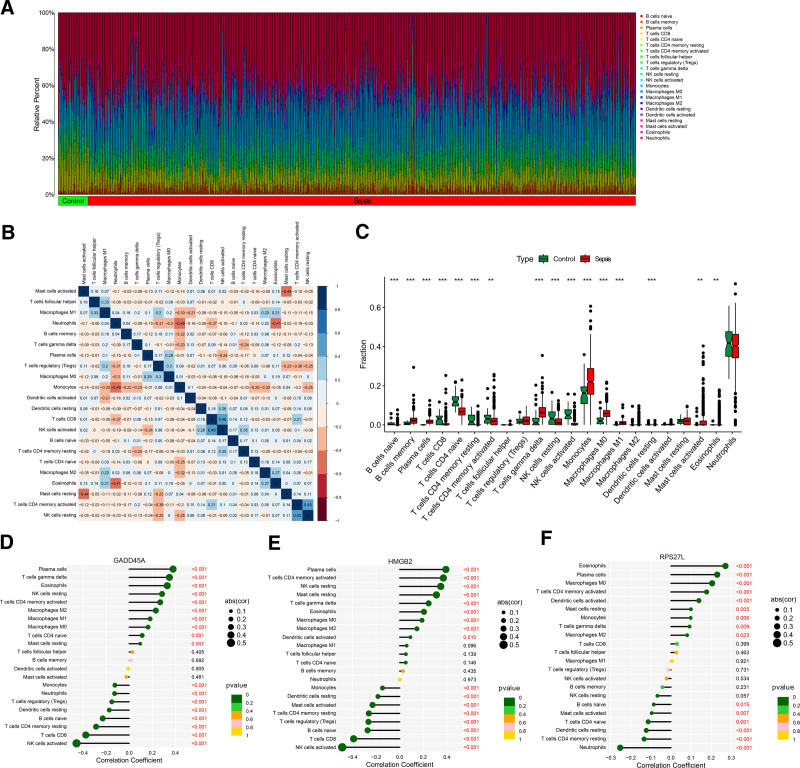
Immune infiltration analysis in septic patients. (A) Bar plot illustrating the percentage of 22 different immune cell types found in sepsis samples and normal samples. (B) Heatmap displaying the association between 22 different types of immune cells. (C) Box plot showing the ratio differentiation of 22 types of immune cells between normal samples and sepsis samples. (D–F) Correlation analysis of 3 critical genes (GADD45A, HMGB2, and RPS27L) with immune cells.

The signature of infiltrating immune subsets was evaluated via the CIBERSORT method, and the relative percent of 22 immune cells in septic patients was calculated to reveal the relationship between the immunological microenvironment and the selected DDR-related genes. Figure 7A and B, respectively, exhibit its makeup in septic patients and the relationships among immune cells. The box plot exhibits the immune cells types infiltration in sepsis and normal samples (Fig. 7C). We observed a significantly higher infiltration of B cells memory, plasma cells, T cells gamma delta, monocytes, macrophages M0, macrophages M1, mast cells activated, and eosinophils in sepsis samples, while the proportions of B cells naive, T cells CD8, T cells CD4 naive, T cells CD4 memory resting, T cells CD4 memory activated, NK cells resting, NK cells activated, and dendritic cells resting were relatively lower. Correlation analysis showed that GADD45A was positively correlated with plasma cells, T cells gamma delta, eosinophils, NK cells resting, T cells CD4 memory activated, macrophages M2, macrophages M1, macrophages M0, T cells CD4 naive, and mast cells resting and negatively correlated with monocytes, neutrophils, T cells regulatory, dendritic cells resting, B cells naive, T cells CD4 memory resting, T cells CD8, and NK cells activated (Fig. 7D). HMGB2 was positively correlated with plasma cells, T cells CD4 memory activated, NK cells resting, mast cells resting, T cells gamma delta, eosinophils, macrophages M0, macrophages M2, and dendritic cells activated and negatively correlated with monocytes, dendritic cells resting, mast cells activated, T cells CD4 memory resting, T cells regulatory, B cells naive, T cells CD8, and NK cells activated (Fig. 7E). RPS27L was positively correlated with eosinophils, plasma cells, macrophages M0, T cells CD4 memory activated, dendritic cells activated, mast cells resting, monocytes, T cells gamma delta, and macrophages M2 and negatively correlated with B cells naive, mast cells activated, T cells CD4 naive, dendritic cells resting, T cells CD4 memory resting, and neutrophils (Fig. 7F).

Taken together, the above results reveal a significant difference between healthy controls and septic patients in the immune microenvironment and closely associated with GADD45A, HMGB2, and RPS27L.

### 3.7. Consensus clustering analysis of 3 selected genes for sepsis

**Figure 8.**
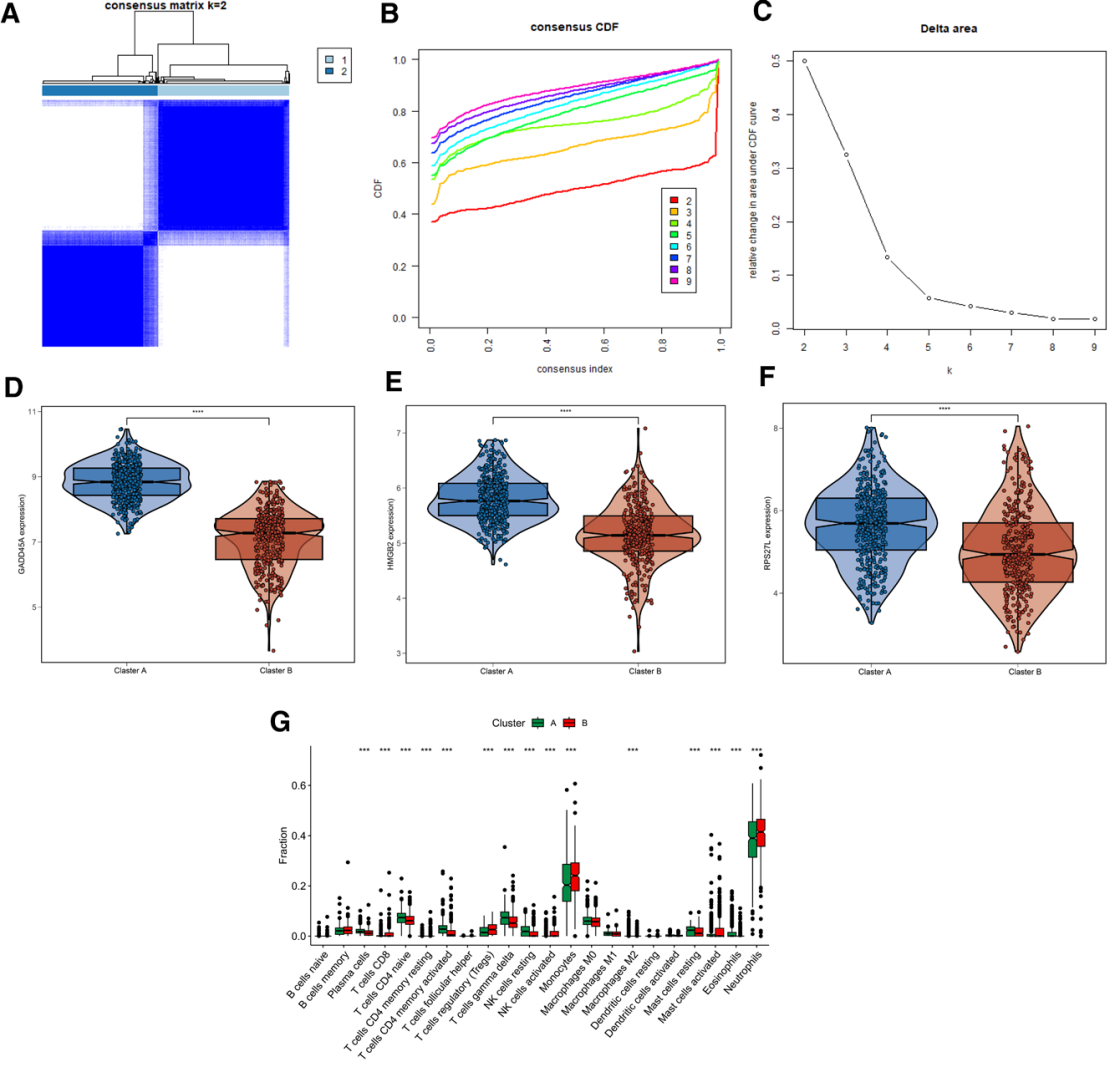
Subgroup analysis of septic patients based on 3 feature biomarkers. (A–C) Consensus clustering analysis. (D–F) The expression of GADD45A, HMGB2, and RPS27L in both cluster subgroups. (G) Immune microenvironment analysis of subgroups.

We carried out a consensus clustering analysis to further cluster the septic patients into different molecular subgroups. According to the 3 selected genes, 2 optimal classifications were acquired for sepsis (Fig. 8A–C). Figure 8D–F showed that the expression of GADD45A, HMGB2, and RPS27L was higher in Cluster A. In addition, the immune microenvironment of samples in Clusters A and B was greatly different (Fig. 8G). These findings indicate that the septic patients could be accurately divided into different molecular subgroups according to the 3 selected genes and significantly related with the immune microenvironment.

## 4. Discussion

Sepsis is a potentially life-threatening systemic inflammatory response syndrome caused by the infiltration of bacteria or toxins in the bloodstream.^[23]^ In recent years, some innovative therapeutic techniques have been applied in clinical. For example, blood purification through the removal or inactivation of endotoxins and inflammatory cytokines has been investigated to determine if it can be used as additional supportive therapy in sepsis. The most studied method is hemoadsorption, in which blood is passed through adsorbent membranes for the removal of endotoxins.^[24]^ Extracorporeal membrane oxygenation provides pulmonary and/or cardiac support for critically ill patients. It releases the failing lung and/or heart from its functions while it is being treated, recovered, or replaced.^[25]^ However, several issues still need to be addressed, such as the cost of extracorporeal membrane oxygenation, therapeutic protocols, initiation time, standardization of indications, and choice of the patients who will benefit most from these interventions. Due to the heterogeneity of individual and the complex pathophysiology of sepsis, the long-term management is not always successful. The Surviving Sepsis Campaign has emphasized that the early diagnosis and prompt treatment of sepsis are important for improving prognosis.^[26]^ However, there are currently no reliable biomarkers for early diagnosing sepsis. DDR system has been reported to play an important role in tumorigenesis, neurodegenerative disorders, immune deficiencies and infertility, stem cell dysfunction, cardiovascular disease, and metabolic syndrome. To our knowledge, no bioinformatics studies investigating the relationship between DDR and sepsis can be retrieved.

Using bioinformatics analysis, 111 DDR-related DEGs were obtained. GO and KEGG analyses revealed that these DEGs were mainly related with double-strand break repair, positive regulation of response to DNA damage stimulus, regulation of chromosome organization, DNA replication, and positive regulation of DNA metabolic process. These findings indicated that DDR-related DEGs play a significant role in sepsis.

Based on these DDR-related DEGs, we used 3 machine learning methods to identify optimal biomarkers of sepsis. The LASSO algorithm defines variables by means of searching for the value corresponding to the lowest likelihood of classification error. The random forest algorithm is a nonparametric technique that could be employed to obtain classification while being supervised. In addition, the SVM algorithm has numerous applications, such as the selection of the most significant ones for classification and the ranking of features. Importantly, we identified 3 pivotal genes, including GADD45A, HMGB2, and RPS27L, which exhibited good diagnostic prediction for sepsis.

So far, these 3 genes have not been thoroughly investigated in sepsis. GADD45A belongs to the GADD45 family and encodes a ubiquitously expressed protein in normal adult and embryonic tissues. It is often induced by DNA damage and other stress signals, and regulates genes involved in growth arrest and apoptosis.^[27]^ Previous studies have reported that knocking down GADD45A can reduce DNA damage-induced apoptosis,^[28,29]^ and it promotes apoptosis by upregulating p38 and JNK.^[30]^ Other studies showed that GADD45A expression levels significantly increased in liver ischemia–reperfusion injury, and it has been proposed as a marker of hepatic ischemia–reperfusion injury.^[31,32]^ Furthermore, GADD45A has been reported to take part in the process of many kidney diseases, such as chronic kidney disease, doxorubicin-induced kidney injury, and resistance to chemotherapy or radiotherapy in renal cell carcinoma cells.^[33–35]^ Our results revealed that the expression of GADD45A was significantly higher in septic patients. Due to the limited number of studies, the role of GADD45A in sepsis remained unclear and our study may provide some insights for future study. HMGB2 has been involved in various cellular processes, such as cell differentiation, DNA replication, repair, transcription, migration, and so on.^[36]^ Previous studies have exhibited that HMGB2 was highly expressed in patients with myocardial infarction and positively correlated with the severity of myocardial infarction and cellular apoptosis.^[37]^ Furthermore, Huang et al^[38]^ found that downregulation of HMGB2 could reduce the infarct size, inflammatory responses, and apoptosis in cerebral injury. However, the role of HMGB2 in septic patients is unknown. RPS27L, an evolutionarily conserved ribosomal protein, plays an important role in maintenance of genome integrity.^[39]^ It is known to play a significant role in cancer, but its role in sepsis is unclear.

Recent studies have revealed that sepsis includes the activation of immune response^[40]^ which is correlated with elevated levels of chemokines,^[41]^ proinflammatory cytokines,^[41]^ proteins released by activated platelets as well as neutrophils, coagulation factors, and complement products.^[42]^ Immune response participates in regulating sepsis sepsis.^[43]^ Studies have shown that immune cells in the early stages of sepsis often exhibit an overreactive state, which gradually develops into immunosuppression phase with the disease progresses.^[44]^ Sepsis immunosuppression makes patients more vulnerable to concurrent infections and organ dysfunction.^[45]^ Immune cell infiltration has been demonstrated to play a significant role in the progression of sepsis.^[46]^ Previous studies revealed that increased apoptosis of CD4+, CD8+, and Th17 lymphocytes,^[47]^ decreased NK cells,^[48]^ and decreased B cells^[49]^ were related with poor prognosis of sepsis. In the present study, the CIBERSORT algorithm was employed to analyze and compare the immune cell infiltration abundance in septic patients and control groups. Significant differences were found in the immune cell infiltration of various immune cells. Furthermore, the expression of GADD45A, HMGB2, and RPS27L were associated with a wide variety of immune cells. These data suggest that these genes may be related to the alterations of immunological microenvironment in patients with sepsis.

There are several limitations that should be acknowledged in our study. Firstly, all the analyses were based on bioinformatics analysis. Further experimental evidence is required to demonstrate the potential function of DDR-related DEGs in sepsis. Secondly, the molecular mechanism of these genes interacting with the immune cells needs to be further investigated in the experimental study. Therefore, to validate our findings in the future, it is necessary to carry out additional experiments both in vitro and in vivo, together with clinical trials.

In conclusion, we used machine learning algorithms to identify 3 key DDR-related genes, GADD45A, HMGB2, and RPS27L, which exhibit a good diagnostic impact on sepsis. The nomogram including these 3 genes also exhibited a good diagnostic value, providing new targets for early diagnosing sepsis. However, additional studies are required to confirm these findings. The study would provide novel insights into the role of DDR in sepsis and serve as a foundation for future research.

## Acknowledgments

The authors would like to thank all participants of this study for their contributions to scientific research.

## Author contributions

**Conceptualization:** Jin Gu, Dong-Fang Wang, Jian-Ying Lou.

**Data curation:** Jin Gu, Dong-Fang Wang, Jian-Ying Lou.

**Formal analysis:** Jin Gu, Dong-Fang Wang.

**Writing – review & editing:** Jin Gu, Jian-Ying Lou.
